# Identification of Rhizospheric Actinomycete *Streptomyces lavendulae* SPS-33 and the Inhibitory Effect of its Volatile Organic Compounds against *Ceratocystis fimbriata* in Postharvest Sweet Potato (*Ipomoea batatas* (L.) Lam.)

**DOI:** 10.3390/microorganisms8030319

**Published:** 2020-02-25

**Authors:** Xuewei Li, Beibei Li, Shurui Cai, Yu Zhang, Mingjie Xu, Chunmei Zhang, Bo Yuan, Ke Xing, Sheng Qin

**Affiliations:** School of Life Science, the Key Laboratory of Biotechnology for Medicinal Plant of Jiangsu Province, Jiangsu Normal University, Xuzhou 221116, China

**Keywords:** *Ipomoea batatas* (L.) Lam., *Streptomyces lavendulae*, volatile organic compound, fumigant, antimicrobial

## Abstract

Black spot disease, which is caused by the pathogenic fungal *Ceratocystis fimbriata*, seriously affects the production of sweet potato and its quality during postharvest storage. In this study, the preliminary identification of the rhizosphere actinomycete strain SPS-33, and its antifungal activity of volatiles *in vitro* and *in vivo* was investigated. Based on morphological identification and phylogenetic analysis of the 16S rRNA gene sequence, strain SPS-33 was identified as *Streptomyces lavendulae.* Volatile organic compounds (VOCs) emitted by SPS-33 inhibited mycelial growth and sporulation of *C. fimbriata* *in vitro* and also induced a series of observable hyphae morphological changes. In an *in vivo* pathogenicity assay, exposure to SPS-33 significantly decreased the lesion diameter and water loss rate in sweet potato tuberous roots (TRs) inoculated with *C. fimbriata*. It increased the antioxidant enzymes’ activities of peroxidase, catalase, and superoxide dismutase as well as decreased malondialdehyde and increased total soluble sugar. In the VOC profile of SPS-33 detected by a headspace solid-phase micro extraction (HS-SPME) and gas chromatography-mass spectrometry (GC-MS), heptadecane, tetradecane, and 3-methyl-1-butanol were the most abundant compounds. 2-Methyl-1-butanol, 3-methyl-1-butanol, pyridine, and phenylethyl alcohol showed strong antifungal effects against *C. fimbriata*. These findings suggest that VOCs from *S. lavendulae* SPS-33 have the potential for pathogen *C. fimbriata* control in sweet potato postharvest storage by fumigant action.

## 1. Introduction

Sweet potato [*Ipomoea batatas* (L.) Lam.] is one of the 10 most important crops in the world. It has the characteristics of low fat, low caloric, and high dietary fiber content. It is also a medicinal plant, which has many important biological activities, such as anti-tumor, antibacterial, and antioxidant factors [1,2]. At present, sweet potato is widely planted in more than 100 countries in the world, of which the largest cultivated area is the continent of Asia. This is followed by Africa and South America. China is the largest sweet potato planting country with an annual planting area of more than 5 million hm^2^, which accounts for about 65% of the world’s total output [3]. Recently, sweet potato has become increasingly popular in China. In particular, sweet potatoes with good taste and a small size sell for a higher price.

Black spot disease, caused by the plant pathogenic fungi *Ceratocystis fimbriata*, frequently occurs during sweet potato production and particularly during postharvest storage. The main sources of infection of black spot disease are seed sweet potatoes tuberous roots (TRs) with pathogens. Diseased seed TRs or infected TRs without symptoms are mixed with healthy TRs. When conditions are suitable, such as when the soil temperature ranges from 15 °C to 30 °C, particularly 23 °C to 27 °C, pathogens infect TRs mainly through wounds or bud eyes. In China, the loss of sweet potato production caused by *C. fimbriata* is 5% to 10% every year, and the loss is as high as 20% to 50% when it is serious, which can lead to the death of sweet potatoes and cause great economic losses to farmers [4]. Therefore, how to effectively prevent and control the pathogen *C. fimbriata* has become one of the important issues in the safe production of sweet potato. Sweet potato TRs can be chilled or frozen below 10 °C. Therefore, they cannot rely on low temperatures alone to resist *C. fimbriata* infection. At present, chemical fungicide is still an important method to prevent and control the black spot disease of sweet potato. It is mainly used in the treatment of sweet potato TRs before postharvest preservation, seed TRs, and sweet potato seedling before transplanting [5]. In addition, new preservatives such as beneficial microorganisms and chitosan also have a potential application value [5,6]. Due to the environmental pollution and harm to human health resulting from chemical pesticides (e.g., carbendazim and sulfur fumigation), it is of great significance to choose a safe and effective method to control postharvest *C. fimbriata* infection.

Volatile organic compounds (VOCs) are volatile compounds with a low molecular weight that are easy to spread in soil and stored products. Although only a small proportion of microbial species have been shown to produce VOCs [7], many studies have found that bacteria and fungi can produce VOCs that have an antimicrobial effect. *Alcaligenes faecalis* N1-4 isolated from the rhizosphere soil of tea plants produced abundant antifungal VOCs, which significantly inhibited the spore germination and mycelial growth of *Aspergillus flavus* [8]. VOCs produced by endophytic fungi *Muscodor brasiliensis* sp. nov. inhibited *Penicillium digitatum* in vitro as well as in oranges [9]. Microbial VOCs also play an important role to promote plant growth and induce systemic resistance against pathogen attack [10]. The greatest advantage of VOCs is that they do not come into direct contact with agricultural products, which is especially suitable for fruits and vegetables whose surface cannot be treated with water-based solvents. There is growing evidence that microbial VOCs may provide an alternative method to protect agriculture products from pathogens and provide conditions for better food quality and safety [11].

Plant symbiotic actinomycetes play an important role in plant growth and development. Some endophytic and rhizospheric actinomycetes can promote plant growth, inhibit phytopathogens, and help plants resist biotic and abiotic stresses [12,13]. *Streptomyces* spp. are the dominant group in the plant rhizosphere. It has been found that rhizospheric *Streptomyces* spp. can produce abundant secondary metabolites, which can directly promote plant growth or inhibit plant pathogens and indirectly benefit the healthy development of plants [14,15,16]. In recent years, plant symbiotic microorganisms that produce VOCs have attracted plenty of attention. It has been found that VOCs produced by endophytic and rhizospheric microorganisms can promote plant growth, inhibit pathogens, and induce plant resistance [17,18,19].

In our previous study, many plant growths promoting rhizobacteria (PGPR) were isolated from the rhizosphere of sweet potato, including many actinomycetes (unpublished data). After antifungal screening, one *Pseudomonas* strain SPS-41 was found to significantly inhibit the growth of *C. fimbriata* in vitro and in vivo by VOCs [20]. This study indicated that there might be other active strains producing VOC against *C. fimbriata* in the rhizosphere of healthy sweet potatoes. The actinobacterium SPS-33 is one *Streptomyces*-like PGP strain that we obtained previously. In order to evaluate whether strain SPS-33 has the potential to control the black spot disease of sweet potatoes, the following work is carried out in this study: (1) assessing the inhibition effect of VOCs produced by SPS-33 against *C. fimbriata*, (2) evaluating the fumigant effect of controlling *C. fimbriata* infection in sweet potato TRs, and (3) identifying the VOC composition of SPS-33 and evaluating the antifungal effect of selected volatile compounds.

## 2. Materials and Methods

### 2.1. Microorganism, Plant Materials, and Chemicals

Strain SPS-33, which is a *Streptomyces*-like actinobacterium, was isolated from the sweet potato rhizosphere soil at the Sweet Potato Research Institute of Chinese Academy of Agricultural Sciences in Xuzhou, Jiangsu, China. SPS-33, which is grown on malt extract-yeast extract agar (ISP 2 medium) at 28 °C, was preserved in our laboratory. *Ceratocystis fimbriata* ACCC30008, obtained from the Agricultural Culture Collection of China, was cultured on potato dextrose agar (PDA) medium at 28 °C. Sweet potato TRs (cv. ‘Tianmu’) with a similar size (13–15 cm length and 5-7 cm width) and a healthy appearance were purchased from a local market (Xuzhou, China). In preparation for the pathogenicity test, the surface of TRs was sterilized with 2% sodium hypochlorite for 2 min, thoroughly washed with tap water, and air-dried at room temperature.

2-Methyl-1-butanol, phenylethyl alcohol, ethyl dodecanoate, ethyl caprylate, ethyl caprate, 2-octanone, 2-nonanone, hexamethylcyclotrisiloxane, decamethylcyclopentasiloxane, tetradecane, hexadecane, heptadecane, and dimethyl sulfoxide were purchased from Aladdin Industrial Corporation (> 98%, Shanghai, China). Acetone and toluene were purchased from Suyi Chemical Reagent Co. Ltd. (> 99%, Shanghai, China). 2-Pentadecanone and pyridine were purchased from Macklin Biochemical Co. Ltd. (> 99%, Shanghai, China). 3-Methyl-1-butanol was purchased from Sinophram Chemical Reagent Co. Ltd. (≥ 98.5%, Shanghai, China). All the purchased commercial compounds are in a liquid state.

### 2.2. Identification of Strain SPS-33

Strain SPS-33 was identified by the combination of morphological observation, 16S RNA gene sequencing, and phylogenetic analysis methods. The colony and cultural characteristics were observed on ISP 2 agar [21]. The morphology of mycelia and the spore chain were observed by light microscopy (SA3300-PL) after being grown on ISP 2 medium for 7 days at 28 °C. Total genomic DNA extraction and PCR amplification of the 16S rRNA gene was carried out, according to the previously published procedures [22]. The 16S rRNA gene sequence alignment and similarity calculation was determined via the EzTaxon-e database (http://eztaxon-e.ezbiocloud.net/). Phylogenetic trees were constructed using the maximum-likelihood [23], maximum-parsimony [24], and the neighbor-joining [25] tree, which makes algorithms by MEGA 6.0 software [26]. The topologies of the trees were evaluated in a bootstrap analysis based on 1000 re-samplings [27]. The complete 16S rRNA gene sequence (1510 bp) of strain SPS-33 has been deposited in the GenBank under the accession number MN540709.

### 2.3. Antagonistic Test of VOCs Emitted by SPS-33

The antagonistic assay of VOCs produced by SPS-33 against *C. fimbriata* was determined according to the previous research [20] with minor modifications. Potato dextrose agar (PDA) medium was added to one side of the I-plates (diameter, 90 mm, volume, 0.07 L), and ISP 2 medium was added to the other side of the same plate. SPS-33 was inoculated on ISP 2 medium by streaking and incubated at 28 °C for 2 days until white aerial mycelium appeared. Then, *C. fimbriata* mycelial discs (5-mm diameter) were inoculated on PDA medium. I-plates with *C. fimbriata* on one half were used as controls. All of the I-plates were completely sealed with Parafilm to form a closed environment and incubated at 28 °C. Two vertical diameters of each *C. fimbriata* colony were measured daily with Vernier calipers until fungal mycelium reached the edge of the control plates. The inhibition rate was determined according to the formula: $\mathrm{inhibition}\;\mathrm{rate}\;\left(\%\right)=\left(1-\frac{Dt-Dd}{Dc-Dd}\right)\times 100\%$, where *Dc* is the average diameter of the mycelial in the control plate, *Dt* is that of the test plate, and *Dd* is that of the inoculated mycelium discs. Meanwhile, the sporulation capacity was counted every day with a hemocytometer. Mycelial morphology was observed after dyeing with lactophenol cotton blue [20].

### 2.4. Pathogenicity Assay

The postharvest in vivo experiment was performed similarly to the previous procedure [6] with minor modifications. Twenty micro-wounds (5-mm deep) were made using the sterile needle tip in the middle of one side of the sweet potato TRs to form a circle with a diameter of about 8 mm. Then, the sweet potato TRs and six ISP 2 agar plates containing 200 μL SPS-33 suspensions (cultured at 28 °C, 120 rpm for 5 days) were sealed in plastic boxes (volume, 0.75 L). Sweet potato TRs without SPS-33 treatment placed in plastic boxes were used as controls. After incubation at 28 °C for 20 days, a series of indicators for disease resistance and quality of sweet potato TRs were evaluated. The weight loss rate of TRs was detected by the weighing method. Evaluation of disease severity (DS) was based on a five-degree empirical scale: (1) 0 mm < lesion diameter ≤ 8 mm, (2) 8 mm < lesion diameter ≤ 16 mm, (3) 16 mm < lesion diameter ≤ 24 mm, (4) 24 mm < lesion diameter ≤ 32 mm, and (5) 32 mm < lesion diameter ≤ 40 mm. DS was calculated according to the formulas in the literature [20].

For antioxidant enzymes and malondialdehyde (MDA) analysis, 1.0 g of the sweet potato TRs was homogenized in 2 mL of 0.1 M phosphate buffer (pH 7.0) on ice. After centrifugation at 10,000 rpm for 20 min, the supernatant was collected for a subsequent detection. Activity of peroxidase (POD) was determined by a guaiacol colorimetric method [28], catalase (CAT) activity was measured spectrophotometrically at 240 nm [29], and superoxide dismutase (SOD) was measured by determining the scavenging degree for the superoxide anion radical using the nitro blue tetrazolium (NBT) method [30]. The MDA content was measured via the thiobarbituric (TBA) reaction on the basis of the previously described procedure [31]. An anthrone method was used to quantify the total soluble sugar (TSS) of sweet potato TRs [32].

### 2.5. Analysis of VOCs Emitted by SPS-33

The VOCs produced by SPS-33 were analyzed by headspace solid-phase micro extraction (HS-SPME) and gas chromatography-mass spectrometry (GC-MS). The extraction and detection method of VOCs were previously reported [16]. SPS-33 was incubated on ISP 2 medium in a headspace vial at 28 °C for 7 days. A blank ISP 2 medium served as the control. The HS-SPME fiber (DVB/CAR/PDMS, Supelco, Bellefonte, PA, USA) was used to extract the volatile compounds at 50 °C for 30 min. The GC–MS analyses were determined by the Agilent 7890B GC system and Agilent 5975C mass spectrometer. The GC-MS condition used for analysis was as mentioned according to the method described by Zhang et al. [20]. The Chroma TOF 4.3X software (LECO Corporation) was used for peaks extraction, filtering and calibration, and VOCs produced by strain SPS-33 were tentatively identified by the National Institute of Standards and Technology (NIST 14) database.

### 2.6. Inhibitory Effect of Selected Volatile Compounds on C. fimbriata

According to the test results of HS-SPME/GC-MS analysis, 15 volatile compounds with a similarity ≥800 and area ≥0.9% were selected for this antifungal assay. Components presented in both the control and SPS-33 treated groups were removed. Different volumes of volatile compounds sterilized by filter membranes were dropped on filter papers. Sterile water served as the control. *C. fimbriata* mycelial discs were placed on the other compartment of I-plates (diameter, 90 mm) containing PDA medium. The inhibition rate was calculated after being incubated at 28 °C for 7 days.

### 2.7. Statistical Analysis

All experiments were carried out at least three times. The data analysis and graphical drawing were performed using the Graphpad Prism 7.0 software, and differences among groups were identified by one-way analysis of variance. The results are expressed as the mean ± standard deviation.

## 3. Results

### 3.1. Identification of Strain SPS-33

**Figure 1:**
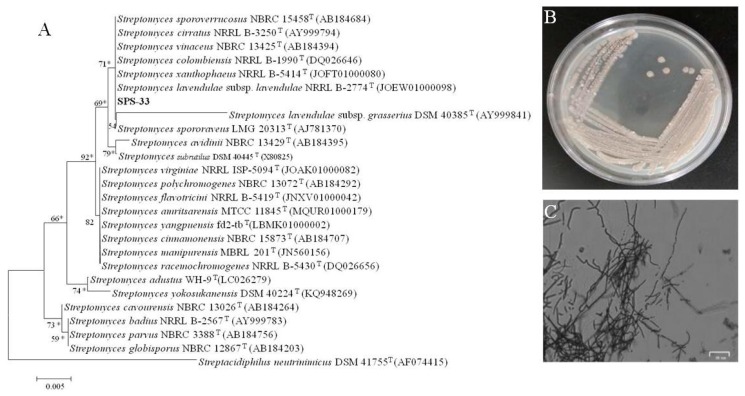
Identification of strain SPS-33. (**A**) The 16S rRNA gene phylogenetic tree constructed by a maximum-likelihood method based on the Kimura 2-parameter model showing the phylogenetic status in the genus *Streptomyces*. Bootstrap values greater than 50% are given at nodes. Asterisks indicate branches of the tree that were also recovered using maximum parsimony and neighbor-joining analysis of 1000 replications. (**B**) Colony morphology traits of strain SPS-33 was grown on ISP 2 medium agar at 28 °C for 7 days. (**C**) Microscopic morphological characteristics of strain SPS-33 showing the mycelia growth at 28 °C for 7 days on ISP 2 medium agar. Scale bar: 25 *μ*m.

Strain SPS-33 grew well on the ISP 2 agar plates, and its colony was loose and grey (Figure 1B). It also formed branched substrate and aerial hyphae, which broke into rods after maturation (Figure 1C). Morphological observation revealed that strain SPS-33 had the typical colony and mycelial characteristics of the genus *Streptomyces*. 16S rRNA gene sequencing and analysis revealed that strain SPS-33 showed 100% sequence identity to *Streptomyces lavendulae*, and they formed a distinct subclade in the phylogenetic tree (Figure 1A). Thus, strain SPS-33 was identified as a new strain of the species *Streptomyces lavendulae.*

### 3.2. Antagonistic Test of VOCs Produced by SPS-33

**Figure 2:**
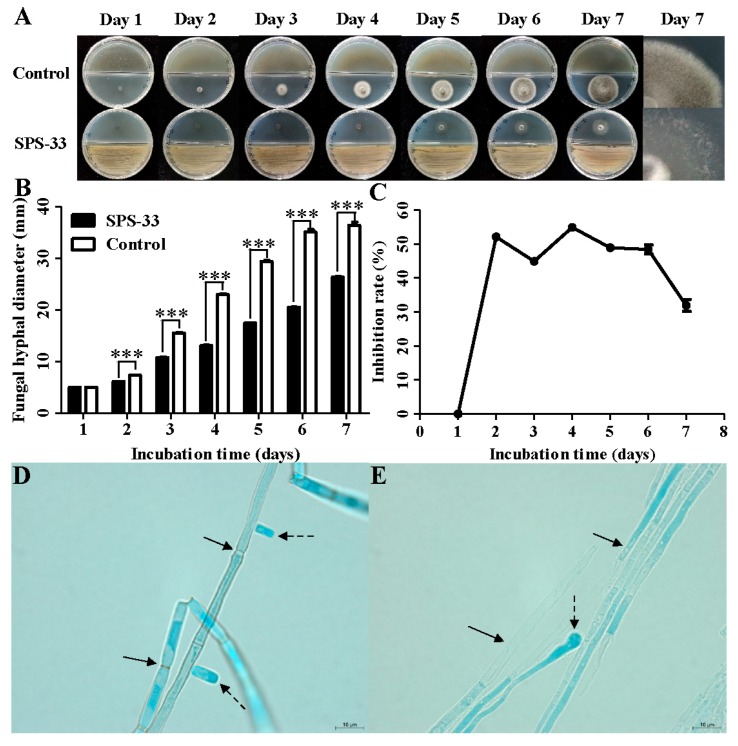
Antifungal effects of VOCs emitted by SPS-33 against *C. fimbriata*. (**A**) Mycelial growth. (**B**) Inhibitory effects on mycelial growth. (**C**) Inhibition rate. (**D**) Morphological observation of untreated *C. fimbriata*. Solid-line arrows point to septum, and dashed-line arrows point to spores. (**E**) Morphological observation of SPS-33 treated *C. fimbriata.* Solid-line arrows point to vacuoles and dashed-line arrows point to abnormal enlargement. Scale bar: 10 μm. *** denotes significance at *p* < 0.001.

In the control group, the colony diameter of *C. fimbriata* increased significantly with the prolongation of culture time. The colony had grown to the edge of the plate on the 7^th^ day (Figure 2A). However, the mycelial growth was significantly inhibited in the SPS-33 treated group. From the second day after inoculation, there was a significant difference in the colony diameter between two groups (Figure 2B). The highest inhibition rate of mycelial growth was 54.92%, which appeared on the fourth day (Figure 2C). Although the inhibition rate did not increase continuously, the morphology of the fungal colonies clearly changed. In the control group, the colonies were dense and coupled with a felt-like appearance, which indicates that the aerial mycelium were abundant. Colonies in the treatment group not only had a smaller size, but also displayed a sparse mycelial distribution (Figure 2A).

It was found that spores of *C. fimbriata* in the control group were produced from the second day after inoculation. The spore production continued to increase with the prolongation of incubation time with a sporulation capacity of 1.14×10^7^ spores/mL. However, *C. fimbriata* in the treatment group did not produce spores within 7 days.

All of the hyphae in the control group were stained by lactophenol cotton blue. The hyphal interiors were fully filled with abundant cellular inclusions. Hyphae were connected together, similar to bamboo and displayed clear and visible septum. Short rod spores were observed around hyphae (Figure 2D). Hyphae treated by SPS-33 were discontinuously stained, which showed many uncolored areas and vacuoles. The septum was difficult to observe, and an abnormal enlargement occurred at the hyphal end (Figure 2E).

### 3.3. Pathogenicity Assay

**Figure 3:**
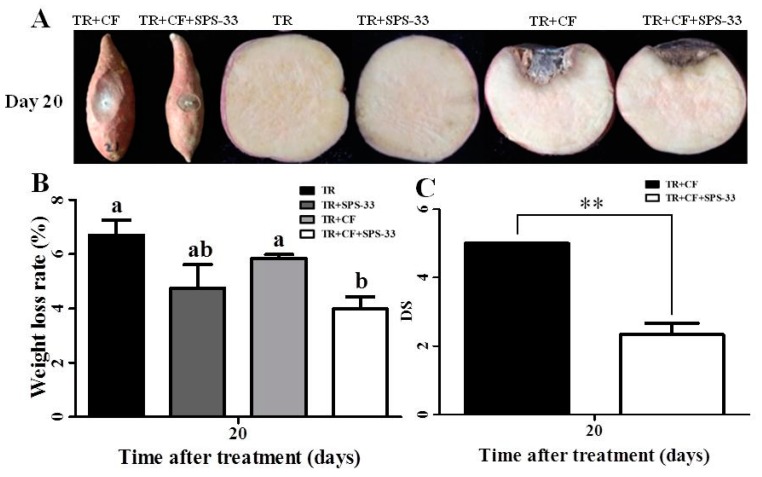
Fumigant effects of SPS-33 in sweet potato TRs. (**A**) Inhibition of black spot disease development in sweet potato TRs. (**B**) Weight loss rate of sweet potato TRs. Means with different letters denote significant differences (*p* < 0.05). (**C**) DS of sweet potato TRs. **denotes significance at *p* < 0.01. TR, tuberous root. CF: *C. fimbriata*. DS: disease severity.

The lesion diameter of the TR+CF group significantly expanded at the end of the 20 days of storage time. In the TR+CF+SPS-33 group, exposure to SPS-33 significantly decreased the lesion diameter caused by *C. fimbriata* compared with the TR+CF group. There were no lesions on the surfaces of the TR group and the TR + SPS-33 group without *C. fimbriata* inoculation. Furthermore, the black spot spread rapidly to the inside of the TRs in the transverse section of the TR + CF group. More than one-third of the transverse section turned black after 20 days. In particular, the surface of TRs sagged inward, and the lesions became dry and empty because of the large area of the lesions. Less lesion areas and milder symptoms were observed in the TR+CF+SPS-33 group. There were no black spots or other symptoms in the transverse section of the TR group and the TR+ SPS-33 group (Figure 3A).

After incubation for 20 days, the weight loss rate of the TR+CF group was significantly higher than that of the TR+CF+SPS-33 group. There was no significant difference in the weight loss rate between the TR group and the TR+ SPS-33 group (Figure 3B). Similar results were observed regarding the DS in Figure 3C. The DS of the TR+CF group was significantly higher than that of the TR+CF+SPS-33 group.

The effects of fumigation on the physiological indicators of TRs were evaluated by antioxidant enzymes and the content of MDA and TSS (Table 1). For antioxidant enzymes activity, there was no significant difference for the TR+SPS-33 groups. The SOD, POD, and CAT activities of the TR+CF+SPS-33 group were significantly higher than that of the control groups after 20 days. The MDA content of the TR+CF+SPS-33 group and the TR+SPS-33 group was significantly lower than the SPS-33 untreated groups. After 20 days of culturing, the TSS contents of the TR+SPS-33 and TR+CF+SPS-33 groups were significantly higher than that of the control.

### 3.4. Analysis of VOCs Emitted by SPS-33

Based on the analysis of VOCs obtained by HS-SPME combined with GC-MS, 20 volatile compounds produced by *S. lavendulae*, SPS-33 with a relative area of more than 0.9% are listed (Table 2). Heptadecane (16.73%) was the most abundant, which was followed by tetradecane (10.84%), 3-methyl-1-butanol (9.40%), acetone (5.41%), and pyridine (5.35%). Fifteen volatile compounds were selected for the antifungal assay, according to their physicochemical properties and antimicrobial activity reports.

### 3.5. Inhibitory Effect of Selected Volatile Compounds on C. fimbriata

As shown in Table 3, 9 of the 15 compounds showed different inhibitory effects on *C. fimbriata*, and their antifungal activities were increased with the increase of volume. 2-Methyl-1-butanol and 3-methyl-1-butanol showed the strongest antifungal activities against *C. fimbriata* with an inhibition rate of 100% at 30 μL/plate concentration. The next was pyridine with an inhibition rate of 100% at 70 μL/plate concentration. Another alcohol, which is phenylethyl alcohol, also had strong antifungal activity against *C. fimbriata* with an inhibition rate of 88.38% at 50 μL/plate concentration. Six of the 15 volatile compounds, i.e., heptadecane, tetradecane, pentadecane, ethyl dodecanoate, hexadecane, and 2-pentadecanone, had no inhibitory effect on *C. fimbriata* at the tested volume range.

## 4. Discussion

The genus *Streptomyces*, as a typical representative of actinomycetes, is known for producing a variety of antibiotics for the benefit of human health. In recent years, it has been found that many strains of streptomycetes also produce VOCs to inhibit fungi growth. Volatiles generated by *S. alboflavus* TD-1 showed wide-spectrum antimicrobial activity against *Fusarium moniliforme* Sheldon, *Aspergillus flavus*, *A. ochraceus*, *A. niger*, and *Penicillum citrinum* [33]. Volatile substances from *S. globisporus* JK-1 inhibited the development of *P. italicum* in vitro as well as on *Citrus microcarpa* [34]. VOCs produced by *S. albulus* NJZJSA2 significantly repressed mycelial growth of *Sclerotinia sclerotiorum* and *F. oxysporum* [35]. However, there is no report about VOCs produced by *S. lavendulae* to date. The original culture of *S. lavendulae* was isolated from soil by Waksman and Curtis in 1916 [36]. Since Waksman and Woodruff isolated streptothricin from it in 1942 [37], numerous reports explored the secondary metabolites and their further functions of *S. lavendulae*, including antibacterial, antifungal, antioxidant, cytotoxic, and antiviral activities [38,39,40,41,42,43]. For decades, the research on *S. lavendulae* that has focused on its fermentation broth. To our knowledge, this is the first report on the VOCs produced by the *S. lavendulae* strain.

In this study, the *C. fimbriata* colony fumigated by *S. lavendulae* SPS-33 was sparse and light in color, which indicated that the development of aerial mycelium was significantly inhibited. Fungal reproduction is mainly accomplished by producing a large number of spores. In artificial media, fungi usually produce asexual spores through the differentiation of aerial hyphae. Therefore, the healthy growth and development of aerial mycelium is directly related to the formation of spores, which explains why no sporulation was observed in the SPS-33 treated group. Dyeing photographs directly proved the damage of VOCs to mycelia. The results suggested that VOCs produced by *S. lavendulae* SPS-33 inhibited spore production by affecting the normal growth and development of mycelia. The fumigation of *S. lavendulae* SPS-33 clearly reduced the lesion diameter and disease severity in sweet potato TRs, and, thus, decreased the weight loss rate due to black spot disease. Oxidative burst is one of the earliest defense reactions in the plant to pathogenic infection, which leads to rapid production of high levels of reactive oxygen species (ROS). Recent studies considered that ROS plays a dual role in plant defense mechanisms [44]. Low concentration of ROS acts as an important signal molecule to participate in cell proliferation, differentiation, apoptosis, and adaptation to stress. However, a high concentration of ROS has strong oxidation ability, which can destroy many biological functional molecules, including the peroxidation of membranes. Antioxidants in plant defense systems, such as SOD, POD, and CAT, can reduce ROS and decrease cell damage [45]. The present results indicated that SOD, POD, and CAT activities were enhanced during the culture time after treatment. The reason may be that it takes time to synthesize antioxidant enzymes and increase the expression of antioxidant enzymes induced by VOCs. The MDA production is one of the indicators for identifying stress injury in plants [46]. Results showed that the fumigation of *S. lavendulae* SPS-33 decreased MDA content, which further verified the detection of antioxidant activities.

TSS is mainly composed of sucrose, glucose, and fructose. The high content of TSS in sweet potato TRs is associated with a sweet taste and good edible quality. Soluble sugar is also an important substance for plants to cope with salt stress, drought, and low temperature, which is related to drought resistance and freeze resistance of sweet potatoes [47]. Therefore, TSS is one of the most important indicators to measure the quality of sweet potatoes. In this study, we found that the TSS content of sweet potatoes fumigated by strain *S. lavendulae* SPS-33 were increased significantly with or without pathogen infection (Table 1). ‘Tianmu,’ which is used in the pathogenicity assay, is a very popular edible-type sweet potato TR known for their good taste in recent years. Fumigation treatment not only alleviated the symptoms of black spot disease, but also increased the TSS contents during storage. Thus, it is significant to maintain the quality of sweet potato during postharvest storage.

As the genus *Streptomyces* produces large quantities of antibiotics, so does its VOC production capacity. Of the 120 VOCs isolated from 26 *Streptomyces* spp. cultured on Emmerson’s yeast starch agar, the most common compounds included isoprene, acetone, 3-methyl-1-butanol, 2-methyl-1-butanol, cyclopentanone, dimethyl disulfide, dimethyl trisulfide, phenylethyl alcohol, and geosmin [48]. It was indicated that some frequently produced VOCs may be the by-products of primary metabolism [49] like an earthy-smelling substance geosmin [34]. However, most of the VOCs are strain-specific, which means each strain produces a unique profile of VOCs [49]. The VOCs produced by streptomycetes generally include alcohols, esters, ketones, sulfur compounds, and terpenoid compounds [48]. As shown in Table 2, the main alcohols produced by *S. lavendulae* SPS-33 were 3-methyl-1-butanol, 2-methyl-1-butanol, and phenylethyl alcohol. These three volatiles have good antimicrobial activity and can be produced by a variety of microorganisms, such as *S. fimicarius* BWL-H1, different *Actinobacteria* isolates, and *Pseudomonas chlororaphis* subsp. *aureofaciens* SPS-41 [20,49,50]. It was found that alcohols showed a noticeably higher antimicrobial activity than esters and ketones in this study, which was consistent with other reports [9,20]. Ethyl decanoate, ethyl dodecanoate, and ethyl octanoate, classified into medium chain fatty acid ethyl esters, were relatively high-content esters produced by *S. lavendulae* SPS-33. These esters are commonly used as additives in the food industry for their excellent aroma characteristics [51]. Ethyl octanoate and ethyl decanoate showed antifungal activity against *C. fimbriata* to some extent, but ethyl dodecanoate did not. In this study, most ketones did not show an inhibition effect on *C. fimbriata*, except acetone. Ketones produced by *Pseudomonas chlororaphis* 449 inhibited the formation of biofilms of *Agrobacterium tumefaciens* and reduced the number of living cells in mature biofilms [52]. Volatile ketones may form Schiff bases with amino groups of proteins, which interferes with the normal functions of proteins [52]. Sulfur compounds and terpenoid compounds were not listed in the table because their content was very low. However, some sulfur VOCs, such as dimethyl disulfide produced by some *Streptomyces* spp., showed significantly antifungal activity against storage pathogens [33]. This indicated that microbially-mediated VOC compositions are affected by many factors, such as strains, growth media composition and proportion, and culture conditions. We noticed that heptadecane and tetradecane, which are the most abundant volatile compounds produced by SPS-33, had no clear inhibition activity against *C. fimbriata*. The following work is to change the VOC composition and improve the antimicrobial ability of *S. lavendulae* SPS-33 by optimizing the culture medium and conditions.

## 5. Conclusions

To our knowledge, this is the first study that focuses on the VOCs produced by *S. lavendulae*. VOCs emitted by *S. lavendulae* significantly inhibited the development of *C. fimbriata in vitro* as well as in postharvest sweet potato TRs. Furthermore, fumigation of SPS-33 caused a series of positive physiological reactions in TRs during storage. These results suggested that *S. lavendulae* SPS-33 could be a potential biological fumigant to control black spot disease in postharvest sweet potato. The fumigation activity of the strain needs to be further optimized and improved in a further study.

## Figures and Tables

**Table 1:** Effect of SPS-33 fumigation on enzyme activities, MDA content, and total soluble sugar content in sweet potato TRs.

20 Days after Treatment	TR	TR+SPS-33	TR+CF	TR+CF+SPS-33
SOD activity (U/g FW)	13.98 ± 3.04 b	15.50 ± 1.79 b	15.39 ± 1.85 b	18.86 ± 0.86 a
POD activity (U/g FW)	2.67 ± 0.67 b	2.89 ± 0.38 b	2.89 ± 0.38 b	4.44 ± 0.38 a
CAT activity (U/g FW)	9.82 ± 1.31 b	8.82 ± 1.04 b	11.43 ± 1.25 b	17.51 ± 2.55 a
Content of MDA (μmol/L)	107.90 ± 5.12 a	77.26 ± 4.52 c	95.84 ± 4.74 b	77.43 ± 10.30 c
Total Soluble sugar (μg/g)	7.78 ± 0.46 d	9.89 ± 0.56 c	11.09 ± 0.15 b	13.16 ± 1.11 a

The results are presented as the mean values ± standard deviation. Means with different letters for each volume (a-d) denote differences (*p* < 0.05).

**Table 2:** HS-SPME/GC-MS analysis of the VOCs produced by *S. lavendulae* SPS-33.

Volatiles Name *^a^*	Retention Time (min)	Area *^b^* (%)	CAS no.	Molecular Formula	Molecular Weight
Heptadecane	25.3799	16.73	629-78-7	C_17_H_36_	240.4677
Tetradecane	17.7886	10.84	629-59-4	C_14_H_30_	198.3900
3-Methyl-1-butanol	12.4138	9.40	123-51-3	C_5_H_12_O	88.1500
Acetone	2.50379	5.41	67-64-1	C_3_H_6_O	58.0800
Pyridine	11.4099	5.35	110-86-1	C_5_H_5_N	79.1000
Pentadecane	20.7159	2.31	629-62-9	C_15_H_32_	212.4100
Ethyl decanoate	23.8919	2.27	110-38-3	C_12_H_24_O_2_	200.3200
2-Methyl-1-butanol	12.3605	1.88	137-32-6	C_5_H_12_O	88.1500
Phenylethyl alcohol	29.7686	1.87	60-12-8	C_8_H_10_O	122.1700
Dodecane	11.5077	1.64	112-40-3	C_12_H_26_	170.3800
2-Butanone	3.36553	1.63	78-93-3	C_4_H_7_O	72.1100
Tridecane	15.1679	1.57	629-50-5	C_13_H_28_	184.4100
Ethyl dodecanoate	28.4849	1.48	106-33-2	C_14_H_28_O_2_	228.3800
Furan	2.37497	1.37	110-00-9	C_4_H_4_O	68.0740
Hexadecane	23.0435	1.37	544-76-3	C_16_H_34_	226.4400
2-Pentadecanone	32.0784	1.35	2345-28-0	C_15_H_30_O	226.4000
2-Octanone	14.426	0.99	111-13-7	C_8_H_16_O	128.2100
Ethyl octanoate	18.7303	0.94	106-32-1	C_10_H_20_O_2_	172.2700
2-Nonanone	17.4421	0.90	821-55-6	C_9_H_18_O	142.2400
Naphthalene	25.9841	0.90	624-92-0	C_10_H_8_	128.1800

*^a^* VOCs with an area of >0.9%. *^b^* The relative area of a single compound is the percentage of the total area of the chromatographic peak.

**Table 3:** Inhibition effects of the VOCs on *C. fimbriata* mycelial growth.

Volatile Compounds	Inhibition Rate (%) of the VOCs at Different Volumes			
	10 μL/plate	30 μL/plate	50 μL/plate	70 μL/plate
2-Methyl-1-butanol	38.18 ± 1.55 b	100.00 ± 0.00 a	100.00 ± 0.00 a	100.00 ± 0.00 a
3-Methyl-1-butanol	33.63 ± 3.38 b	100.00 ± 0.00 a	100.00 ± 0.00 a	100.00 ± 0.00 a
Phenylethyl alcohol	25.59 ± 0.52 c	44.05 ± 1.11 b	88.38 ± 1.06 a	88.72 ± 0.92 a
Pyridine	14.70 ± 1.50 d	41.75 ± 0.47 c	86.36 ± 0.29 b	100.00 ± 0.00 a
Ethyl octanoate	6.06 ± 0.76 c	10.44 ± 0.38 c	23.35 ± 0.47 b	46.41 ± 1.72 a
Ethyl decanoate	4.32 ± 1.61 c	13.97 ± 0.52 b	20.87 ± 0.77 a	24.19 ± 0.61 a
2-Octanone	1.57 ± 0.08 d	8.02 ± 0.46 c	18.74 ± 0.26 b	23.29 ± 0.70 a
2-Nonanone	7.74 ± 0.67 c	16.22 ± 0.21 b	21.71 ± 3.55 b	43.77 ± 0.71 a
Acetone	10.66 ± 0.75 c	15.21 ± 0.82 c	23.51 ± 0.60 b	28.68 ± 0.65 a

The results are presented as the mean values ± standard deviation. Means with different letters for each volume (a-d) denote differences (*p* < 0.05).

## Abbreviations

TRs	tuberous roots,
VOC	volatile organic compound,
PDA	potato dextrose agar,
POD	peroxidase,
CAT	catalase,
SOD	superoxide dismutase,
MDA	malondialdehyde,
TSS	total soluble sugar,
HS-SPME	headspace solid-phase microextraction,
GC-MS	gas chromatography-mass spectrometry,
ROS	reactive oxygen species,
CF	*C. fimbriata*,
DS	disease severity.
